# miR-449a inhibits cell proliferation, migration, and inflammation by regulating high-mobility group box protein 1 and forms a mutual inhibition loop with Yin Yang 1 in rheumatoid arthritis fibroblast-like synoviocytes

**DOI:** 10.1186/s13075-019-1920-0

**Published:** 2019-06-03

**Authors:** Yongsong Cai, Congshan Jiang, Jialin Zhu, Ke Xu, Xiaoyu Ren, Lin Xu, Peijing Hu, Bo Wang, Qiling Yuan, Yuanxu Guo, Jian Sun, Peng Xu, Yusheng Qiu

**Affiliations:** 1 Department of Orthopaedics of the First Affiliated Hospital, Xi’an Jiaotong University Health Science Center, Xi’an, 710061 China; 2 Department of Joint Surgery, Xi’an Honghui Hospital, Xi’an Jiaotong University Health Science Center, Xi’an, 710054 China; 3 Department of Biochemistry and Molecular Biology, School of Basic Medical Sciences, Xi’an Jiaotong University Health Science Center, Xi’an, 710061 China; 4 Department of Orthopaedics of the 3201 Hospital, Hanzhong, 723000 China; 5 Department of Cardiovascular Medicine of the Second Affiliated Hospital, Xi’an Medical School, Xi’an, 710038 China; 6 Center for Translational Medicine, The First Affiliated Hospital of Xi’an Jiaotong University Health Science Center, Xi’an, 710061 China

**Keywords:** miR-449a, Rheumatoid arthritis, Fibroblast-like synoviocytes, Proliferation, Inflammation, High-mobility group box protein 1

## Abstract

**Background:**

We previously found that high-mobility group box protein 1 (HMGB1) promoted cell proliferation, migration, invasion, and autophagy in rheumatoid arthritis fibroblast-like synoviocytes (RA-FLS), but little is known about its regulatory mechanism. The aim of this study was to investigate the regulatory mechanism of HMGB1 at the posttranscription level.

**Methods:**

Real-time qPCR, CCK-8 cell proliferation assay, transwell cell migration assay, enzyme-linked immunosorbent assay (ELISA), and western blotting were used in this study. The targeting relationship between miRNA and mRNA was presented by the luciferase reporter assay.

**Results:**

MiR-449a was downregulated in RA synovial tissue and inhibited RA-FLS proliferation, migration, and IL-6 production. MiR-449a directly targeted HMGB1 and inhibited its expression. Yin Yang 1(YY1) negatively regulated miR-449a expression and formed a mutual inhibition loop in RA-FLS. MiR-449a inhibited TNFα-mediated HMGB1 and YY1 overexpression and IL-6 production.

**Conclusions:**

Our results reveal the regulatory mechanism of HMGB1 in RA and demonstrate that miR-449a is a crucial molecule in RA pathogenesis and a suitable candidate for miRNA replacement therapies in RA.

**Electronic supplementary material:**

The online version of this article (10.1186/s13075-019-1920-0) contains supplementary material, which is available to authorized users.

## Background

Rheumatoid arthritis (RA) is a chronic autoimmune inflammatory disease characterized by synovial hyperplasia and joint destruction [1]. The understanding of the pathogenic mechanisms of RA and treatments for RA have been greatly improved in the last decade, but there is still no cure for RA [2]. Accumulating evidence indicates that RA fibroblast-like synoviocytes (RA-FLS) play a key role in the pathogenesis of RA [3].

Studies have found that microRNAs (miRNAs) control various cellular processes and play an important role in different diseases, including RA [4, 5]. Recently, several miRNAs were observed to be abnormally expressed in RA, and some of which affected RA-FLS cell proliferation, inflammation, or cellular signaling pathways [6–8]. MiR-449a, located on chromosome 5q11, has been reported to be dysregulated in some diseases and influences cell apoptosis, proliferation, invasion, migration, and inflammation by regulating the expression of various genes [9–14]. Our previous research found that miR-449a expression was downregulated in RA synovial tissue compared with osteoarthritis (OA) tissue. However, little is known about the function of miR-449a in RA.

High-mobility group box protein 1 (HMGB1) is a highly conserved chromatin-binding nuclear protein that has been confirmed to play important roles in many diseases [15–17]. Our previous studies found that HMGB1 expression was increased in RA synovial tissue and that HMGB1 promoted cell proliferation, migration, invasion, and autophagy in RA-FLS [18, 19]. Therefore, exploring the molecular mechanisms of HMGB1 dysregulation in RA may help us better understand the pathogenesis of RA. HMGB1 has been reported to be regulated by miRNAs in some diseases [20–24], but whether the overexpression of HMGB1 in RA is related to dysregulated miRNAs remains unknown.

In the present study, we showed that miR-449a expression was downregulated in RA synovial tissue. miR-449a inhibited RA-FLS proliferation, migration, and IL-6 production by targeting HMGB1 and Yin Yang1 (YY1). YY1 negatively regulated miR-449a expression and formed a mutual inhibition loop in RA-FLS. We further found that miR-449a inhibited TNFα-mediated HMGB1 and YY1 overexpression and IL-6 production. miR-449a may be a crucial molecule in RA pathogenesis and a suitable candidate for miRNA replacement therapies in RA.

## Methods

### Patients and tissue collection

Human synovial tissue samples were obtained from 14 OA patients and 14 RA patients undergoing knee arthroplasty surgery (Department of Joint Surgery, Xi’an Hong Hui Hospital, Xi’an, China). This study was performed with the approval of the human research ethics committee of Xi’an Hong Hui Hospital. All patients fulfilled the American College of Rheumatology(ACR) criteria for the classification of RA or OA and provided informed consent. Information on all patients is summarized in Table 1.

**Table 1:** Patient characteristics

Clinical data	RA	OA
Number of patients	14	14
Sex		
Male	4	6
Female	10	8
Age^†^ (years)	59.4 ± 8.2	68.9 ± 4.9
Disease duration^†^ (years)	9.3 ± 2.3	15.1 ± 3.5
RF^†^ (IU/ml)	123.3 ± 112.2	7.1 ± 3.5
ESR^†^ (mm/h)	41.9 ± 12.9	22.9 ± 17.3
CRP^†^ (mg/l)	27.6 ± 15.4	9.6 ± 9.2
Anti-CCP positive, *n*	12/14	0/14

*RF* rheumatoid factor, *ESR* erythrocyte sedimentation rate, *CRP* C-reactive protein, *CCP* cyclic citrullinated peptide

^†^Mean ± standard deviation

### Cell culture

FLS were dissociated from RA synovial tissue specimens, and 3rd to 8th passage cells were used in our study. The cells were grown in DMEM/F12 medium (Thermo Fisher Scientific, Waltham, MA, USA) supplemented with 10% fetal bovine serum (Gibco Life Technologies, USA), 100 units/mL penicillin, and 100 μ g/mL streptomycin at 37 °C under 5% CO_2_.

### Oligonucleotides and vector construction

The miR-449a mimics, YY1-siRNA targeting human YY1, and negative control RNAs for both the miRNA and siRNA were chemically synthesized by GenePharma (GenePharma, Shanghai, China). For the expression vector, HMGB1 and YY1 were cloned into pCMV-Blank (Beyotime, Shanghai, China) between the site BamH1 and site EcoR1. We used a bioinformatics analysis to determine that both HMGB1 and YY1 are targets of miR-449a (TargetScan, www.targetscan.org). The linker fragment containing the HMGB1 or YY1 wild-type or the mutant 3′UTR-binding site was synthesized and cloned into the pmirGLO Dual-Luciferase vector (Promega, Madison, WI, USA) between site Sac1 and site Xho1. All the primer sequence information is given in Additional file 1: Table S1.

### RNA extraction and quantitative reverse transcription PCR

Total RNA was isolated from the synovial tissue samples and cells using TRIzol reagent (Invitrogen; Thermo Fisher Scientific, Inc.) according to the manufacturer’s protocol. Two micrograms of total RNA and a Transcriptor cDNA Synthesis Kit (Thermo Fisher Scientific, Waltham, MA, USA) were used to synthesize cDNA, which was subsequently used to detect mRNA expression. A total of 500 ng of total RNA and mir-XmiRNA First-Strand Synthesis Kits (Clontech, Mountain View, CA, USA) were used to synthesize cDNA, which was subsequently used to detect miRNA expression. qPCR using a SYBR Green System (Roche Diagnostics, Mannheim, Germany) was used to detect gene expression. β-actin and U6 expression levels were used as endogenous controls to normalize mRNA and miRNA expression, respectively. The forward primer for miR-449a was purchased from Tiangen Biotech (Tiangen Biotech, Beijing, China), and the reverse primer was obtained from the miR-X miRNA First-Strand Synthesis Kits. The other primers used in this study are listed in Additional file 1: Table S1. The 2^−ΔΔCT^ method was used to quantify the relative expression.

### Cell proliferation assay

Cell proliferation under different stimulation conditions was assessed using a cell counting kit-8 (Nanjing KeyGen Biotech, Co., Ltd., Nanjing, China) according to the manufacturer’s protocol.

### Cell migration assay

Cell migration under different stimulation conditions was performed using a Transwell assay (Sigma Company, St. Louis, Missouri, USA) according to Mu’s protocol [25], with some minor modifications. Briefly, after undergoing transfection for 48 h, RA-FLS were starved in serum-free DMEM/F12 medium for another 24 h. Then, then 3 × 10^4^ cells were plated in 200 μl of serum-free medium in the upper chamber with the noncoated membrane (8 μm pores), and the lower chamber was filled with 500 μl of medium containing 10% FBS. After incubating for 24 h at 37 °C, the cells on the upper surface of each membrane were removed with a cotton swab, and those on the bottom side of the membrane were stained with crystal violet and counted under a microscope.

### Enzyme-linked immunosorbent assay (ELISA)

After RA-FLS were stimulated for 48 h, the cell culture supernatants were collected and diluted for the measurement of IL-6 secretion by ELISA (BOSTER, Wuhan, China) according to the manufacturer’s recommendations.

### Cell transfection

RA-FLS were transfected with the miRNA (20 nM), siRNA (20 nM), negative control RNA (20 nM), or expression vectors encoding HMGB1 (1 μg/ml) or YY1 (1 μg/ml) using Lipofectamine 2000 Transfection Reagent (Invitrogen; Thermo Fisher Scientific, Inc.) according to the manufacturer’s recommendations.

### Luciferase reporter assay

HEK293 cells were cotransfected with 100 ng pmirGLO vectors containing the wild-type or mutant 3′UTR of HMGB1 or YY1 mRNA and 20 nM of miR-449a or 20 nM of the miRNA negative control according to the manufacturer’s protocol. After 24 h, luciferase activity was assayed using a Dual-Luciferase Reporter Assay System Kit (Promega, Madison, WI, USA). The relative firefly luciferase activity was normalized to the Renilla luciferase activity.

### Western blotting

A 20-μg protein sample was separated by sodium dodecyl sulfate-polyacrylamide gel electrophoresis (SDS-PAGE) and then electrophoretically transferred to nitrocellulose membranes (Millipore, Billerica, MA, USA). After blocking in 10% nonfat dry milk in Tris-buffered saline/Tween-20 (TBST) for 2 h, the membranes were then incubated with the following primary antibodies overnight at 4 °C: anti-β-actin monoclonal antibody (Bioss, Beijing, China, 1:1000), anti-HMGB1 monoclonal antibody (Abcam, Cambridge, UK, 1:2000), and anti-YY1 monoclonal antibody (Abcam, Cambridge, UK, 1:1000). After washing three times in TBST for 10 min each time, the membranes were incubated with goat anti-rabbit horseradish peroxidase-conjugated secondary antibodies (BOSTER, Wuhan, China, 1:10000) for 1 h at room temperature. After that, the membranes were washed three times with TBST and detected using an electrochemiluminescence (ECL) system (Gene Gnome 5, Synoptics Ltd., UK). The protein levels were normalized to the β-actin protein levels in each sample.

### Statistical analysis

Each experiment was repeated three times, and the data were presented as the mean ± standard deviation (SD) or mean with 95% confidence interval (CI). Differences between two groups were analyzed using Student’s *t* test, or the Mann-Whitney *U* test for experiments in which the datasets were not normally distributed. All the results were analyzed with GraphPad Prism 5.0 software (GraphPad Software, La Jolla, CA, USA). *P* values less than 0.05 were considered statistically significant.

## Results

### MiR-449a inhibits cell proliferation, migration, and inflammation in RA-FLS

**Fig. 1:**
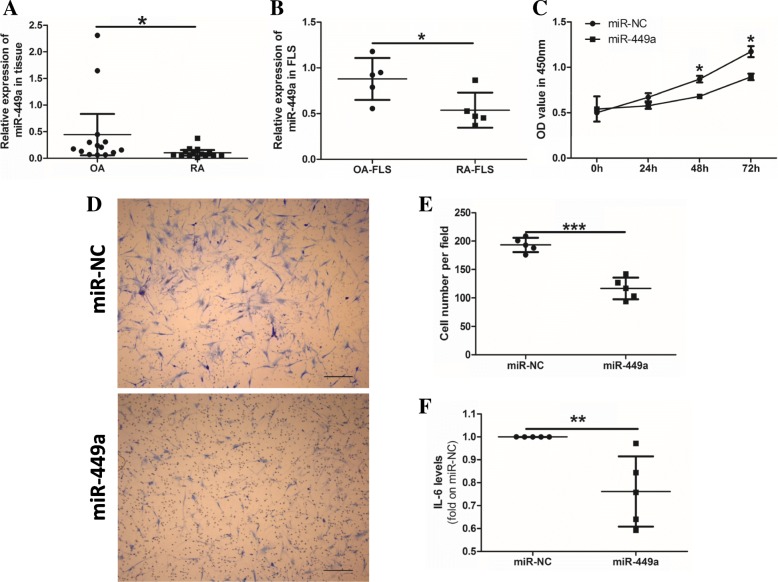
miR-449a inhibits cell proliferation, migration, and IL-6 production in RA-FLS. miR-449a expression in synovial tissue samples (14 RA and 14 OA) and FLS (5 RA-FLS and 5 OA-FLS) was detected using qPCR (**a**, **b**). Cell proliferation was detected via a CCK-8 assay (**c**). Cell migration was assessed by a Transwell assay using RA-FLS transfected with miR-449a or miR-NC. Scale bar: 50 μm (**d**, **e**). The IL-6 production in cell culture supernatants was detected by ELISA (**f**, *n* = 5). The results are representative of three experiments using cells from different patients. U6 snRNA was used as the endogenous control for the miRNA qPCR, and β-actin was used as the endogenous control for the mRNA qPCR. The data were analyzed by the Mann-Whitney test (**a**, **b**, and **f** ). Student’s *t* test was used for the data analyses (**c**, **e**). *p < 0.05, ***p < 0.001. The data was expressed as the mean with 95% CI, the other data expressed as the mean ± SD (**a**)

The quantitative PCR (qPCR) results indicated that miR-449a expression was significantly downregulated both in RA synovial tissue and RA-FLS compared with OA (Fig. 1a, b). RA-FLS were transfected with 20 nM miR-449a mimic or 20 nM negative control RNA (miR-NC) for 24 h, 48 h, or 72 h. Cell proliferation was detected via a CCK-8 assay. The optical density (OD) value showed that RA-FLS in the miR-449a mimic groups had lower cell proliferation ability than those in the miR-NC groups (Fig. 1c). We also detected the effect of miR-449a on cell migration and IL-6 production in RA-FLS. Both cell migration (Fig. 1d, e) and IL-6 production (Fig. 1f) were significantly inhibited in the RA-FLS transfected with the miR-449a mimic compared with the miR-NC groups. These data demonstrated that miR-449a negatively regulated cell proliferation, migration, and IL-6 production in RA-FLS.

### HMGB1 is a direct target of miR-449a in RA

**Fig. 2:**
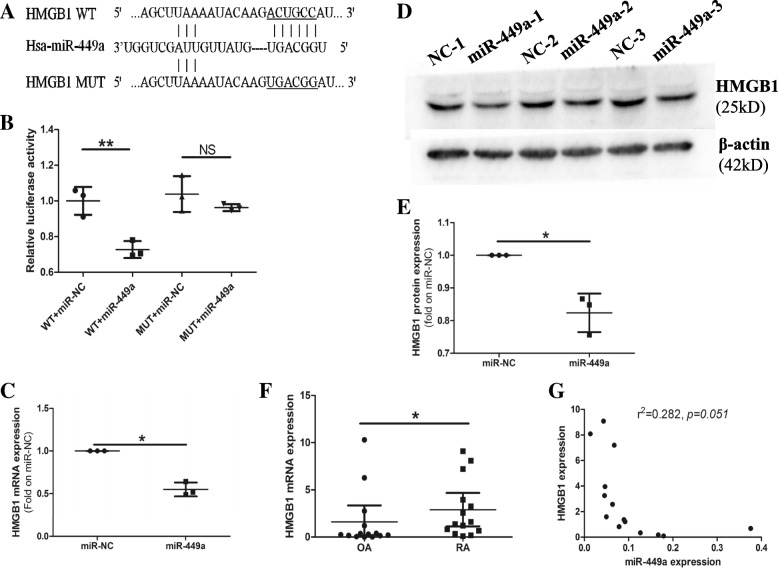
HMGB1 is a direct target of miR-449a in RA-FLS. **a** A putative miR-449a-binding site exists in the 3′UTR of HMGB1 mRNA. **b** A Dual-Luciferase Reporter Assay was performed to validate the target relationship between miR-449a and HMGB1. The results represent three independent experiments. After transfecting miR-449a into RA-FLS, HMGB1 expression was detected by qPCR (**c**) and western blotting (**d**, **e**). The data represent three experiments using cells from different patients. HMGB1 expression in RA and OA patient synovial tissue samples was detected by qPCR (**f**). The correlation between miR-449a and HMGB1 was analyzed (**g**). U6 snRNA was used as the endogenous control for the miRNA qPCR, and β-actin was used as the endogenous control for the mRNA qPCR and western blotting. Student’s *t* test was used for the data analyses (**b**, **e**). Student’s *t* test with Welch’s correction was used for the data analysis in (**c**). The Mann-Whitney test was used for the data analysis in (**f**). The linear regression analysis was used for the data analysis in (**g**). *p < 0.05, **p < 0.01. Data (**f**) expressed as the mean with 95% CI, the other data expressed as the mean ± SD

Based on the bioinformatics prediction, we identified a putative binding site for miR-449a in the HMGB1 mRNA 3′UTR (Fig. 2a). The Dual-Luciferase Reporter Assay results showed that miR-449a inhibited the luciferase activity in the cells containing the miR-449a-binding site of the HMGB1 3′UTR but failed to affect the cells containing a mutated version of the miR-449a-binding site of the HMGB1 3′UTR (Fig. 2b), which indicated that miR-449a bound directly to the 3′UTR of the HMGB1 mRNA to suppress translation. After the transfection of RA-FLS with the miR-449a mimic or miR-NC, the expression of HMGB1 was detected. The results revealed that miR-449a inhibited HMGB1 expression at both the mRNA (Fig. 2c) and protein levels (Fig. 2d, e).

qPCR results showed that the expression of HMGB1 was significantly increased in RA synovial tissue compared with OA tissue (Fig. 2f). Immunohistochemistry results also found that HMGB1 was overexpressed in RA synovial tissue (Additional file 2: Figure S1). Furthermore, the analysis of the expression of miR-449a and HMGB1 mRNA in RA synovial tissue indicated an inverse correlation between the two (Fig. 2g), which suggested that miR-449a might be an important regulator of HMGB1 expression in RA. All the results indicated that HMGB1 was a direct target of miR-449a in RA-FLS.

### Overexpression of HMGB1 eliminates the effects of miR-449a in RA-FLS

**Fig. 3:**
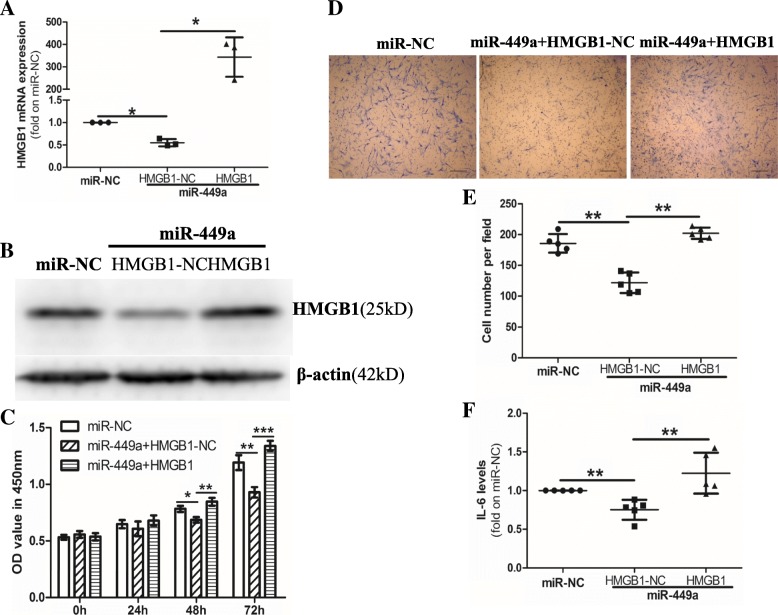
Overexpression of HMGB1 eliminates the effects of miR-449a on cell proliferation, migration, and IL-6 production in RA-FLS. RA-FLS were transfected with miR-NC or cotransfected with miR-449a and the HMGB1 expression vector (HMGB1) or HMGB1 negative control vector (HMGB1-NC) for the indicated times. The expression of HMGB1 was detected using qPCR (**a**) and western blotting (**b**). Cell proliferation was detected via a CCK-8 assay (**c**). Cell migration was performed in transwells. Scale bar: 50 μm. (**d**, **e**). IL-6 production was detected by ELISA (**f**, *n* = 5). Data expressed as the mean ± SD, β-actin was used as the endogenous control for the mRNA qPCR and western blotting. One-way ANOVA was used for data analysis. *p < 0.05, **p < 0.01, ***p < 0.001

Given that HMGB1 promotes cell proliferation, migration, invasion, and autophagy in RA-FLS, we wanted to demonstrate whether miR-449a inhibits cell proliferation, migration, and IL-6 production in RA-FLS by inhibiting the expression of HMGB1. After the transfection of RA-FLS with the HMGB1 expression vector (pCMV-HMGB1), the HMGB1 levels were markedly increased at both the mRNA and protein levels (Fig. 3a, b). After the transfection of RA-FLS with the miR-NC or cotransfection of RA-FLS with the miR-449a and pCMV-HMGB1 (miR-449a + HMGB1) or pCMV blank vector (miR-449a + HMGB1-NC), cell proliferation, migration, and IL-6 production were detected. Compared with the miR-NC group, the miR-449a + HMGB1-NC group inhibited RA-FLS proliferation, migration, and IL-6 production, but the overexpression of HMGB1 reduced the effects of miR-449a in RA-FLS (Fig. 3c–f). These data indicated that the miR-449a-mediated inhibition of RA-FLS proliferation, migration, and IL-6 production might be achieved by inhibiting HMGB1 expression.

### miR-449a and YY1 form a mutual inhibition loop in RA-FLS

**Fig. 4:**
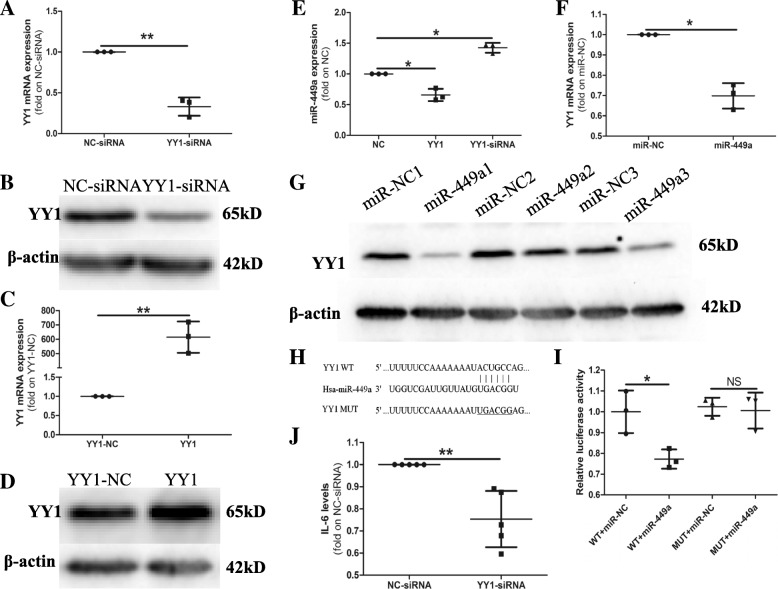
miR-449a and YY1 form a mutual inhibition loop in RA-FLS. RA-FLS were transfected with the YY1-siRNA, negative control siRNA (NC-siRNA), YY1 expression vector (YY1), or YY1 negative control vector (YY1-NC). The expression of YY1 was detected by qPCR (**a**, **c**) and western blotting (**b**, **d**). The expression of miR-449a was detected using qPCR (**e**). qPCR and western blotting were performed to detect the expression of YY1 after the transfection of miR-449a or miR-NC (**f**, **g**). **h** A putative miR-449a-binding site exists in the 3′UTR of YY1 mRNA. A Dual-Luciferase Reporter Assay was performed to validate the target relationship between miR-449a and YY1 (**i**). The IL-6 production in cell culture supernatants was detected by ELISA (**j**, *n* = 5). The results shown are the mean ± SD from three independent experiments. Student’s *t* test with Welch’s correction was used for the data analyses (**a**, **c**, **f**, and **j**). One-way ANOVA was used for the data analysis (**e**). Student’s *t* test was used for the data analysis in (**i**). *p < 0.05, **p < 0.01

Previous studies found that the transcription factor YY1 was closely related to RA inflammation [25, 26]. YY1 regulates IL-6 transcription in RA and inhibits miR-10a expression, which contributes to the excessive secretion of inflammatory cytokines and the migration and proliferation of RA-FLS [25, 26]. Therefore, we sought to determine whether the dysregulation of miR-449a in RA was also related to YY1. The results showed that the YY1 expression markedly decreased at both the mRNA and protein levels (Fig. 4a, b) after the transfection of RA-FLS with the YY1-siRNA. We also induced YY1 expression using the pCMV-YY1 vector (Fig. 4c, d). After the induction or inhibition of YY1 expression, we detected the levels of miR-449a. As shown in Fig. 4e, compared with the negative control, the YY1-siRNA promoted the expression of miR-449a, and pCMV-YY1 (YY1) inhibited miR-449a expression, indicating that YY1 negatively regulates the expression of miR-449a.

Furthermore, we discovered that the overexpression of miR-449a decreased the level of YY1 at both the mRNA and protein levels (Fig. 4f, g). A luciferase activity assay showed that miR-449a inhibited the luciferase activity in cells containing the miR-449a-binding site of the YY1 3′UTR but failed to inhibit the luciferase activity in cells containing a mutated version of the miR-449a-binding site of the YY1 3′UTR (Fig. 4h, i), indicating that YY1 was a direct target gene of miR-449a. These results indicated that miR-449a and YY1 formed a mutual inhibition loop in RA-FLS. We also detected the effect of YY1 on IL-6 production in RA-FLS. As shown in Fig. 4j, the YY1-siRNA inhibited IL-6 production in RA-FLS.

### miR-449a inhibits TNFα-mediated HMGB1 and YY1 overexpression and IL-6 production

**Fig. 5:**
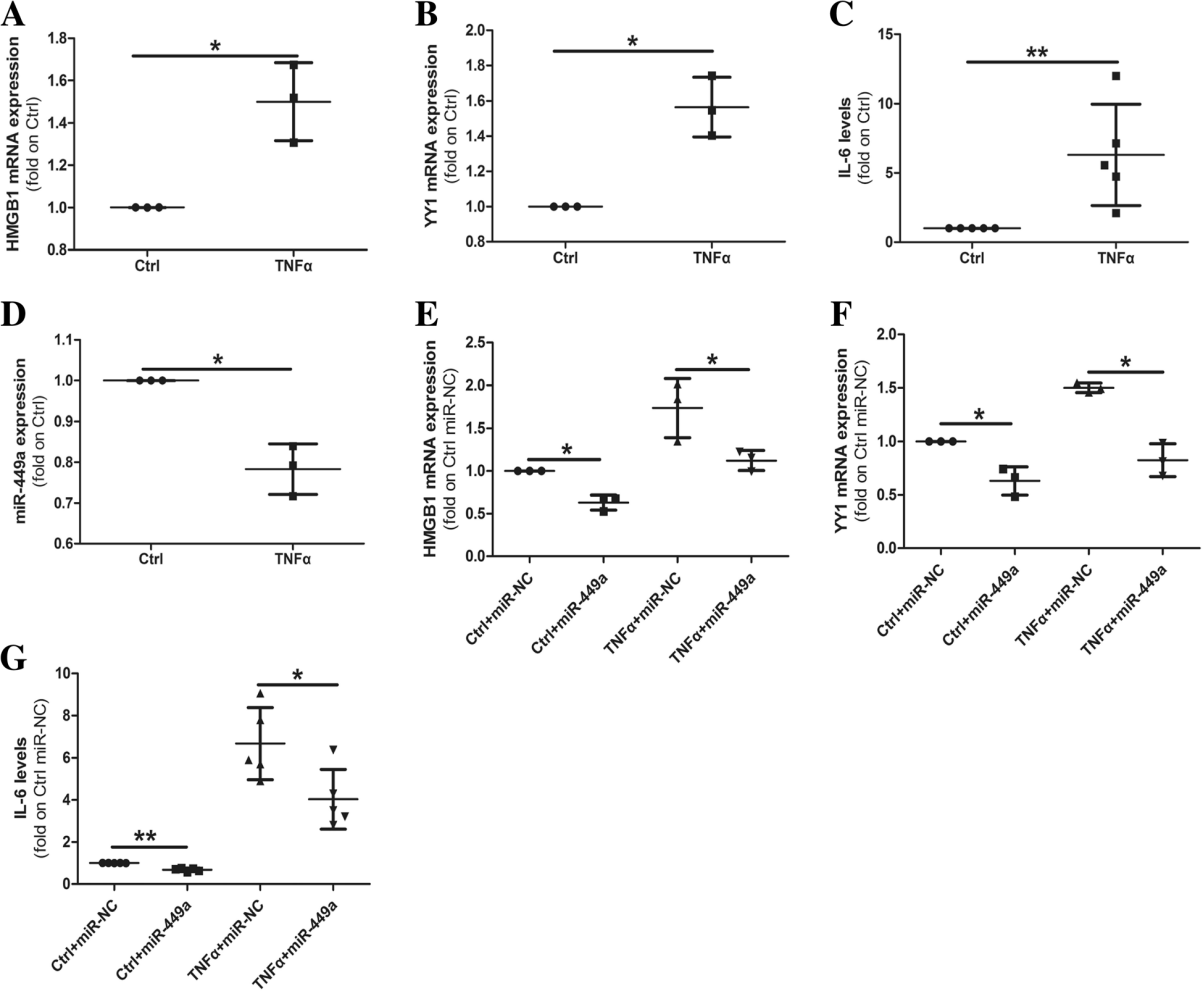
miR-449a inhibits TNFα-mediated HMGB1 and YY1 overexpression and IL-6 production. RA-FLS were treated with 10 ng/ml TNFα or the bovine serum albumin negative control (Ctrl) for 48 h, HMGB1, YY1, and miR-449a expression was detected by qPCR (**a**, **b**, **d**), and IL-6 production was detected by ELISA (**c**, *n* = 5). After RA-FLS cells were treated with 10 ng/ml TNFα in the presence or absence of 20 nM miR-449a for 48 h, HMGB1 and YY1 expression was detected by qPCR (**e**, **f**), and IL-6 production was detected by ELISA (**g**, *n* = 5). The results shown are the mean ± SD from three independent experiments using cells from different patients. Student’s *t* test with Welch’s correction was used for data analysis. *p < 0.05, **p < 0.01

TNFα plays a crucial role in RA pathogenesis. Therefore, we investigated whether TNFα affected HMGB1, YY1 and miR-449a expression and IL-6 production, and the role of miR-449a in this process. After RA-FLS were treated with 10 ng/ml TNFα for 48 h, both the expression of HMGB1 (Fig. 5a) and YY1 (Fig. 5b) and IL-6 production (Fig. 5c) were significantly increased compared with the control-treated RA-FLS, and the expression of miR-449a was decreased(Fig. 5d). We then detected the effects of TNFα in the presence or absence of miR-449a. Interestingly, miR-449a expression weakened TNFα-induced HMGB1 (Fig. 5e) and YY1 (Fig. 5f) expression and IL-6 production (Fig. 5g). These results indicated that miR-449a inhibited TNFα-mediated HMGB1 and YY1 overexpression and IL-6 production.

## Discussion

In the present study, we determined that the expression of miR-449a was significantly decreased in RA synovial tissue compared with OA tissue. miR-449a inhibited cell proliferation, migration, and IL-6 production by regulating HMGB1and YY1 expression in RA-FLS. Further study indicated that YY1 negatively regulated miR-449a expression and formed a mutual inhibition loop with miR-449a in RA-FLS. In addition, we also found that TNFα promoted HMGB1 and YY1 expression and IL-6 production, and miR-449a weakened these effects of TNFα.

Although RA is the most common chronic inflammatory autoimmune joint disease, the pathogenesis of RA remains unclear. RA-FLS, located in the synovial lining, have been proven to play a key role in the RA process [27]. Similar to tumor cells, RA-FLS are a type of special tumor-like cells that are hyperplastic, invasive, and migratory, and these features are closely related to synovial hyperplasia, joint inflammation, and joint destruction [3, 28, 29]. Therefore, we can treat RA as a special tumor-like disease. miRNAs have been proven to be key regulators that target multiple genes in various disease development and progression processes [30, 31]. Several miRNAs were observed to be abnormally expressed in RA, some of which affect RA-FLS cell proliferation, inflammation, or cellular signaling pathways [6–8]. In this study, we detected miR-449a expression and found that it was decreased in RA synovial tissue, which was consistent with the results of some studies in tumors [10, 13, 14, 32, 33]. We further studied the effect of miR-449a on RA-FLS and found that miR-449a inhibited RA-FLS cell proliferation, migration, and IL-6 production, which indicated that the dysregulation of miR-449a expression may be related to RA progression.

Previous studies found that HMGB1 was overexpressed in RA synovial tissue promoted RA-FLS proliferation, migration, and invasion, and HMGB1-mediated autophagy decreased the sensitivity of RA-FLS to methotrexate (MTX) [18, 19]. It seems that HMGB1 plays a nonnegligible role in the pathogenesis of RA. In our study, we found that HMGB1 was regulated by miR-449a and was a direct target of miR-449a in RA-FLS, which was consistent with the study by Wu et al. [24]. They found that miR-449a inhibited cell proliferation, migration, and invasion in non-small cell lung cancer by directly targeting HMGB1. The results indicated that decreased miR-449a expression may be the main cause of HMGB1 overexpression in RA synovial tissue.

YY1 is a transcription factor with different biological functions that can either activate or repress gene expression by directly binding to the target promoters and recruiting histones and DNA modifiers [26, 34]. Recent studies have found that YY1 is overexpressed in RA patients and CIA mice [25, 26]. Mu et al. [25] identified that miR-10a, a YY1 target gene, was inhibited by YY1, which contributed to excessive NF-κB-mediated inflammatory cytokine secretion and RA-FLS migration and proliferation. Lin et al. [26] also found that YY1 promoted IL-6 transcription by directly binding to the IL-6 promoter, which contributed to RA inflammation. These studies indicated that YY1 plays an important role in RA pathogenesis. In this study, when we inhibited the expression of YY1 using an siRNA, we found that miR-449a expression was increased, and when we overexpressed YY1 in RA-FLS, the expression of miR-449a was decreased. The results indicated that YY1 negatively regulated miR-449a expression. Further study found that YY1 was also a direct target of miR-449a. Taken together, the results indicated that miR-449a and YY1 formed a mutual inhibition loop in RA-FLS. We also found that YY1 inhibited IL-6 production in RA-FLS, which was consistent with Lin’s research [26].

TNFα is a key inflammatory cytokine implicated in RA pathogenesis, and it plays a crucial role in joint destruction [35]. In recent years, the biological disease-modifying antirheumatic drugs targeting TNFα (infliximab, adalimumab) have achieved good results in clinical application, but more than half of RA patients still have no responses to the drug or unwanted side effects and eventually have to discontinue the treatment [36, 37]. Therefore, exploring the mechanism of TNFα in RA may contribute to overcoming these limitations. Mu et al. [25] found that an NF-κB/YY1/miR-10a/NF-κB regulatory circuit, which promotes excessive NF-κB-mediated inflammatory cytokine secretion and RA-FLS cell proliferation and migration, existed. In this study, we observed that TNFα promoted HMGB1 and YY1 mRNA expression and IL-6 production. After the transfection of miR-449a, the expression of HMGB1 and YY1 and the production of IL-6 were decreased. These results indicated that miR-449a acted as a regulator to inhibit TNFα-mediated HMGB1 and YY1 overexpression and IL-6 production.

## Conclusions

We first demonstrated that miR-449a inhibited RA-FLS proliferation, migration, and IL-6 production by directly targeting HMGB1 and YY1. miR-449a inhibited TNFα-mediated YY1 and HMGB1 overexpression and IL-6 production and formed a mutual inhibition loop with YY1 in RA-FLS, which indicated that miR-449a was a crucial molecule in RA pathogenesis and acted as a switch to control TNFα-mediated inflammation. As a result, miR-449a-based therapeutic strategies may offer a new treatment option for RA. Further study is needed to confirm the new option in in vivo experiments.

## Additional files

Additional file 1: **Table S1.** Sequences of oligonucleotides. (DOCX 12 kb)

Additional file 2: **Figure S1.** HMGB1 was overexpressed in RA synovial tissue compared with OA tissue. (DOCX 488 kb)

## Data Availability

All the data generated and analyzed during this study are included in this published article and are available from the corresponding author on reasonable request.

## Abbreviations

ACR: American College of Rheumatology
CI: Confidence interval
ELISA: Enzyme-linked immunosorbent assay
HMGB1: High-mobility group box protein 1
miRNAs: MicroRNAs
MTX: Methotrexate
OA: Osteoarthritis
RA: Rheumatoid arthritis
RA-FLS: Rheumatoid arthritis fibroblast-like synoviocytes
SD: Standard deviation
YY1: Yin Yang 1
