# Supplementary material for: miR-449a inhibits cell proliferation, migration, and inflammation by regulating high-mobility group box protein 1 and forms a mutual inhibition loop with Yin Yang 1 in rheumatoid arthritis fibroblast-like synoviocytes
Source: Arthritis Res Ther. 2019 Jun 3;21:134. doi: 10.1186/s13075-019-1920-0 (PMC6547523; doi:10.1186/s13075-019-1920-0)
Supplement: Supplementary file 1 — Table S1. Sequences of oligonucleotides. (DOCX 12 kb) [file 13075_2019_1920_MOESM1_ESM.docx]

**Table S1 Sequences of Oligonucleotides**

| **Name** | **Sense Strand/Sense Primer (5'-3')** | **Antisense Strand/Antisense Primer (5'-3')** |
| --- | --- | --- |
| **Primers for miRNA** | | |
| miR-449a | TGGCAGTGTATTGTTAGCTGGT | mRQ 3’Primer |
| U6 | CTCGCTTCGGCAGCACA | AACGCTTCACGAATTTGCGT |
| **Primers for real-time qPCR** | | |
| HMGB1 | GGAGAGTAATGTTACAGAGCGG | AGGATCTCCTTTGCCCATGT |
| β-actin | ATCGTGCGTGACATTAAGGAGAAG | AGGAAGGAAGGCTGGAAGAGTG |
| YY1 | AGAAGAGCGGCAAGAAGAGTT | CAACCACTGTCTCATGGTCAATA |
| **Primers for cloning (Restriction enzyme sites were underlined)** | | |
| HMGB1-CDS | CGCGGATCCATGGGCAAAGGAGATCCT | CCGGAATTCTTATTCATCATCATCATCTTC |
| YY1-CDS | CGCGGATCC ATGGCCTCGGGCGACACC | CCGGAATTCTCACTGGTTGTTTTTGGCCTTAGCAT |
| HMGB1 3’-UTR WT | CTAGCGGCCGCTAGTAGCTTAAAATAC  AAGACTGCCATC | TCGAGATGGCAGTCTTGTATTTTAAGCTACTAGC  GGCCGCTAGAGCT |
| HMGB1 3’-UTR MUT | CTAGCGGCCGCTAGTAGCTTAAAATACA  AGTGACGGATC | TCGAGATCCGTCACTTGTATTTTAAGCTACTAGC  GGCCGCTAGAGCT |
| YY1 3’-UTR WT | CTAGCGGCCGCTAGTTTTTTCCAAAAAAA  TACTGCCAGC | TCGAGCTGGCAGTATTTTTTTGGAAAAAACTAGC  GGCCGCTAGAGCT |
| YY1 3’-UTR MUT | CTAGCGGCCGCTAGTTTTTTCCAAAAAAA  TTGACGGAGC | TCGAGCTCCGTCAATTTTTTTGGAAAAAACTAGC  GGCCGCTAGAGCT |
| **miRNA** | | |
| miR-449a | UGGCAGUGUAUUGUUAGCUGGU | ACCAGCUAACAAUACACUGCCA |
| miR-NC | UUCUCCGAACGUGUCACGUTT | ACGUGACACGUUCGGAGAATT |
| **siRNA** | | |
| YY1-siRNA（1675） | CCUGAAAUCUCACAUCUUATT | UAAGAUGUGAGAUUUGAGGTT |
| YY1-siRNA（905） | GACGACUACAUUGAACAAATT | UUUGUUCAAUGUAGUCGUCTT |
| YY1-siRNA（1316） | GCUAGAAUGAAGCCAAGAATT | UUCUUGGCUUCAUUCUAGCTT |
| NC-siRNA | UUCUCCGAACGUGUCACGUTT | ACGUGACACGUUCGGAGAATT |
