# Supplementary material for: miR-449a inhibits cell proliferation, migration, and inflammation by regulating high-mobility group box protein 1 and forms a mutual inhibition loop with Yin Yang 1 in rheumatoid arthritis fibroblast-like synoviocytes
Source: Arthritis Res Ther. 2019 Jun 3;21:134. doi: 10.1186/s13075-019-1920-0 (PMC6547523; doi:10.1186/s13075-019-1920-0)
Supplement: Supplementary file 2 — Figure S1. HMGB1 was overexpressed in RA synovial tissue compared with OA tissue. (DOCX 488 kb) [file 13075_2019_1920_MOESM2_ESM.docx]

**Figure S1**

**Materials and methods**

**Immunohistochemistry**

Immunohistochemistry was performed as described elsewhere[^1^](#_ENREF_1), with some minor modifications. Synovial tissue sections were deparaffinized in xylene and rehydrated in graded ethanol. Then, 1% H_2_O_2_ was used to inhibit endogenous peroxidase activity. For antigen retrieval, the sections were heated in a microwave oven in 10 mM sodium citrate buffer (pH 6.0). The sections were blocked with 5% goat serum (Beyotime Institute of Biotechnology, Shanghai, China) for 30 min, and then an anti-HMGB1 antibody (Abcam, Cambridge, UK, 1:200) and the negative control FBS were applied to the sections and incubated at 4 °C overnight. Then, the sections were incubated with a horseradish peroxidase-conjugated goat anti-rabbit antibody (Beyotime, Shanghai, China) at room temperature for 30 min, stained with DAB (Beyotime, Shanghai, China) and mounted.

**Figure S1. HMGB1 was overexpressed in RA synovial tissue compared with OA tissue.**

HMGB1 expression in RA and OA patient synovial tissue samples was detected by immunochemistry. 5% bovine serum albumin was used as negative control (NC).Scale bar:50μm.

**
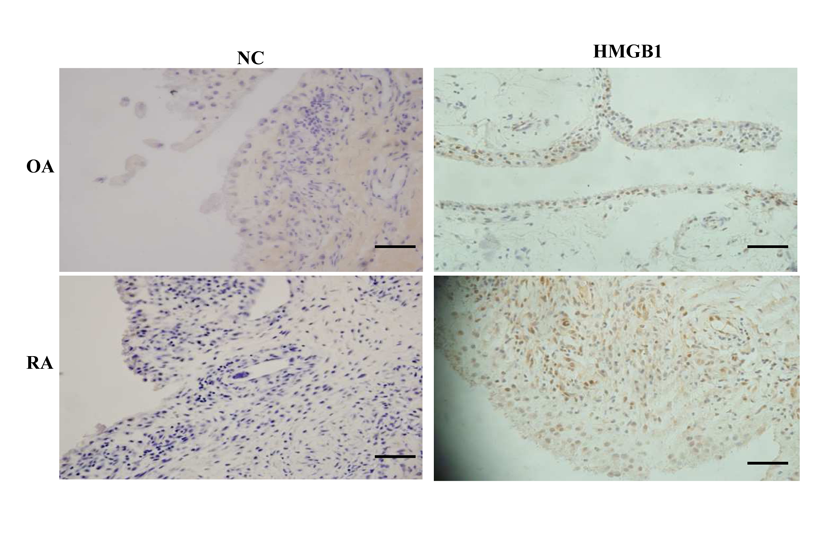
**

**1. Li YB, Xu P, Xu K, et al. Methotrexate affects HMGB1 expression in rheumatoid arthritis, and the downregulation of HMGB1 prevents rheumatoid arthritis progression. *Mol Cell Biochem*. 2016;420(1-2):161-170.**
